# Health-related Quality of Life with Adjuvant Nivolumab After Radical Resection for High-risk Muscle-invasive Urothelial Carcinoma: Results from the Phase 3 CheckMate 274 Trial

**DOI:** 10.1016/j.euo.2022.02.003

**Published:** 2022-03-11

**Authors:** Johannes Alfred Witjes, Matthew D. Galsky, Jürgen E. Gschwend, Edward Broughton, Julia Braverman, Federico Nasroulah, Mario Maira-Arce, Xiaomei Ye, Ling Shi, Shien Guo, Melissa Hamilton, Dean F. Bajorin

**Affiliations:** a Radboud University, Nijmegen, The Netherlands; b Tisch Cancer Institute, Icahn School of Medicine at Mount Sinai, New York, NY, USA; c Technical University of Munich, Munich, Germany; d Bristol Myers Squibb, Princeton, NJ, USA; e Evidera, Waltham, MA, USA; f Memorial Sloan Kettering Cancer Center, New York, NY, USA

**Keywords:** Adjuvant, Bladder cancer, Immunotherapy, Invasive, Nivolumab, Phase 3, Quality of life, Radical cystectomy, Randomized controlled trial

## Abstract

**Background:**

The programmed death-1 (PD-1) inhibitor nivolumab prolongs disease-free survival in patients with muscle-invasive urothelial carcinoma (MIUC).

**Objective:**

To evaluate the effects of nivolumab on health-related quality of life (HRQoL) after radical resection in patients with MIUC.

**Design, setting, and participants:**

We used data from 709 patients in CheckMate 274 (NCT02632409; 282 with programmed death ligand 1 [PD-L1] expression ≥1%), an ongoing randomized, double-blind, placebo-controlled phase 3 trial of adjuvant nivolumab.

**Intervention:**

Intravenous injection of nivolumab (240 mg) or placebo every 2 wk for ≤1 yr.

**Outcome measurements and statistical analysis:**

HRQoL was assessed using the European Organisation for Research and Treatment of Cancer Quality of Life Questionnaire (EORTC QLQ-C30) and the EQ-5D-3L. Linear mixed-effect models for repeated measures were used to compare nivolumab and placebo on changes in HRQoL. Time to confirmed deterioration (TTCD) of HRQoL was analyzed by Cox proportional hazards regression.

**Results and limitations:**

In the full HRQoL evaluable population, no clinically meaningful deterioration of HRQoL was observed in either treatment arm. Moreover, nivolumab was noninferior to placebo on changes from baseline for all main outcomes. The median TTCD for fatigue was 41.0 wk for nivolumab and 44.3 wk for placebo (hazard ratio [HR]: 1.11, 95% confidence interval [CI], 0.89–1.39). For the visual analog scale, the median TTCD was not reached for nivolumab and it was 57.6 wk for placebo (HR: 0.78, 95% CI, 0.61–1.00). The median TTCD for the other main outcomes was not reached in either treatment arm. The findings were similar for patients with PD-L1 expression ≥1%.

**Conclusions:**

These results demonstrate that nivolumab did not compromise the HRQoL of patients with MIUC in CheckMate 274.

## Patient summary

Nivolumab is being researched as a new treatment for patients with bladder cancer (urothelial carcinoma). We found that nivolumab maintained quality of life while increasing the time until cancer returns in patients whose bladder cancer had spread or grown and who had unsuccessfully tried platinum-containing chemotherapy.

## 1. Introduction

Urothelial carcinoma is an immunogenic malignancy originating in the urinary bladder or upper urinary tract (renal pelvis or ureter). Tumors that invade the muscle wall of the bladder are typically high grade, with a high potential for metastasis [1]. Standard of care treatment for muscle-invasive urothelial carcinoma (MIUC) is cisplatin-based neoadjuvant chemotherapy (NAC) followed by radical resection with curative intent [2]. Only 13–39% of patients with muscle-invasive bladder cancer have been reported to receive cisplatin-based NAC [3–6], and usage is even lower in patients with tumors arising in the upper urinary tract [7]. This low utilization is partly because many patients are ineligible for or refuse cisplatin-based chemotherapy [8,9]. Even when patients receive NAC, nearly 30% do not complete their regimen, for reasons including age, comorbidities, and toxicity [10]. Moreover, the risk of recurrence is high [11,12]. Adjuvant chemotherapy within 90 d of resection has been explored, but its utility is limited by patients being ineligible for or refusing cisplatin and having complications of resection [8,13–16]. Therefore, additional effective treatment options are needed.

Nivolumab is a fully human IgG4 monoclonal antibody that binds to programmed death-1 (PD-1). It is approved for patients with locally advanced or metastatic urothelial carcinoma previously treated with platinum-based chemotherapy [17,18], and is now being explored in other indications. In the ongoing phase 3 CheckMate 274 trial of nivolumab in patients with MIUC who have undergone radical resection with or without NAC, median disease-free survival (DFS, primary endpoint) was significantly longer in patients who received nivolumab than in those who received placebo [19].

Previous studies have found that health-related quality of life (HRQoL) can be impaired in patients with MIUC [20], those with muscle-invasive bladder cancer treated with NAC [21], and those who have undergone radical resection for urothelial cancer [22–24]. Any clinical benefit of nivolumab treatment should, therefore, not be compromised by worsening of HRQoL due to treatment-related toxicities. Moreover, while recurrence of MIUC is associated with poor survival [25], its effect on HRQoL is unclear [26].

The main aim of this study was to evaluate the effects of nivolumab on HRQoL in patients who have undergone radical resection of MIUC, using data from CheckMate 274. Another aim was to analyze the association between disease recurrence and deterioration of HRQoL.

## 2. Patients and methods

### 2.1. Study design

The present analysis was based on the phase 3 CheckMate 274 trial (NCT02632409), a randomized, double-blind, placebo-controlled trial of adjuvant nivolumab [19]. For each participating site, approval for CheckMate 274 was obtained from an institutional review board or ethics committee. All patients provided written informed consent.

### 2.2. Patients

Eligible patients were adults (≥18 yr) who had undergone radical resection within the previous 120 d of MIUC originating in the bladder or upper urinary tract (renal pelvis or ureter) and who had a high risk of recurrence based on pathologic stage: ypT2-pT4a or ypN+ for patients who had received cisplatin-based NAC, and pT3-pT4a or pN+ for patients who had not received cisplatin-based NAC and were ineligible for or refused adjuvant cisplatin-based chemotherapy. Full eligibility criteria are available in a different publication [19].

### 2.3. Treatment

Patients were randomized 1:1 to adjuvant nivolumab or placebo. The randomization was stratified by tumor programmed death ligand 1 (PD-L1) expression (≥1% vs <1% or indeterminate), pathologic nodal status (N+ vs Nx or N0 with fewer than ten nodes removed vs N0 with ten or more nodes removed), and use of cisplatin-based NAC (yes vs no).

Patients received nivolumab (240 mg) or placebo by intravenous injection every 2 wk until disease recurrence, unacceptable toxicity, or withdrawal of consent. The maximum treatment duration was 1 yr. After discontinuing study treatment, patients were followed up for survival and recurrence. Recurrence was classified as local only (any new lesion[s] in the lower or upper urothelial tract, or in the pelvic soft tissue or pelvic nodes below the aortic bifurcation) or distant (any new lesion[s] at another site, with or without local recurrence).

### 2.4. Patient-reported outcome assessments

HRQoL, symptoms, and health status were assessed using the European Organisation for Research and Treatment of Cancer Quality of Life Questionnaire (EORTC QLQ-C30) and the EQ-5D-3L. Assessments were completed on cycle 1 day 1 (baseline), every other cycle (every 4 wk) for the first 6 mo of treatment, and then every third cycle (every 6 wk) thereafter until discontinuation of study treatment. Additional assessments were completed at two post-treatment follow-up visits. The first follow-up visit was approximately 35 d after the last dose of study treatment, and the second follow-up visit was approximately 80 d after the first follow-up visit. HRQoL outcomes were among the exploratory end points in the primary CheckMate 274 trial and are the principal outcomes in this analysis.

The EORTC QLQ-C30 includes 30 items across 15 domains: a two-item global health status/quality of life (QoL) domain, five multi-item functional domains (physical functioning, role functioning, cognitive functioning, emotional functioning, and social functioning), three multi-item symptom domains (fatigue, pain, and nausea/vomiting), and six single-item domains (dyspnea, insomnia, appetite loss, constipation, diarrhea, and financial difficulties) [27]. In accordance with the EORTC QLQ-C30 scoring manual [27], a domain score was calculated if responses were given for at least 50% of the items in the domain; otherwise, the score was considered to be missing. Raw scores were standardized through linear transformation to a 0–100 scale. A higher score for global health status/QoL represents better overall HRQoL, a higher score for a functional domain represents a better level of functioning, and a higher score for a symptom domain represents worse symptomatology or problems [27].

The EQ-5D-3L is a self-administered questionnaire where respondents answer five questions on different aspects of their current health (mobility, self-care, usual activities, pain/discomfort, and anxiety/depression) and indicate their overall health on a visual analog scale (VAS) ranging from 0 (worst health imaginable) to 100 (best health imaginable) [28].

The main HRQoL analysis examined five prespecified outcomes: the EORTC QLQ-C30 global health status/QoL, physical functioning, role functioning, and fatigue domains, and the VAS.

### 2.5. Statistical analyses

Analyses were performed with SAS version 9.4 or higher (SAS Institute Inc., Cary, NC, USA) using data collected up to August 27, 2020. The analyses were performed using the EORTC QLQ-C30 evaluable population (patients with a non-missing score for at least one of the EORTC QLQ-C30 domains at both baseline and at least one postbaseline visit) and the VAS evaluable population (patients who had a non-missing VAS score at both baseline and at least one post-baseline visit). Additional analyses were based on patients with tumor PD-L1 expression ≥1%. None of the analyses were adjusted for multiple comparisons.

Summary statistics were calculated for demographics and baseline clinical characteristics, and for patient-reported outcome (PRO) assessments. For PRO assessments, the extent of missing data over time was assessed by calculating the percentage of evaluable assessments using both the number of patients who were still on study (variable denominator rate) and the full intent-to-treat (ITT) population (all randomized patients; fixed denominator rate) as the denominator [29]. Missing data were not imputed.

Within-patient clinically meaningful changes were prespecified using responder definition thresholds, while minimally important differences (MIDs) were prespecified to interpret whether a within-group mean score change or a between-group difference in the mean score change was clinically meaningful. The responder definitions and within-group MIDs were defined as a change from baseline of ±10 points for each domain of the EORTC QLQ-C30 [30] and 7 points for the VAS [31]. Noninferiority of nivolumab versus placebo was assessed using the MID thresholds reported by Cocks and colleagues [32].

Linear mixed-effect models for repeated measures (MMRMs) were calculated using data from assessments during treatment and (for patients who completed 1 yr of treatment) at two post-treatment follow-up visits. The models used a restricted maximum likelihood estimation method, with an unstructured covariance matrix to obtain the random-effect variance components. Score change from baseline was the dependent variable, and treatment, visit, stratification factors, and baseline PRO score were included as covariates. These models were used to estimate least squares (LS) mean changes from baseline in HRQoL scores in each treatment arm. These were also used to estimate differences between nivolumab and placebo in LS mean changes from baseline in HRQoL scores across all visits.

Time to confirmed deterioration (TTCD) of HRQoL (worsening above the responder definition threshold for at least two consecutive visits) was analyzed by the Kaplan-Meier product limit method [33]. Hazard ratios (HRs) for confirmed deterioration of HRQoL for nivolumab versus placebo were estimated using Cox proportional hazards regression models that included the treatment arm and baseline PRO score as covariates, and that were stratified by the same factors as for the randomization. Cox proportional hazards regression was also used to estimate HRs for confirmed deterioration of HRQoL for recurrence (local only, distant, or any) versus no recurrence. The models, which included recurrence as a time-dependent covariate, controlled for the treatment arm and baseline PRO score, and were stratified by the randomization factors.

## 3. Results

### 3.1. Patients

**Fig. 1 –:**
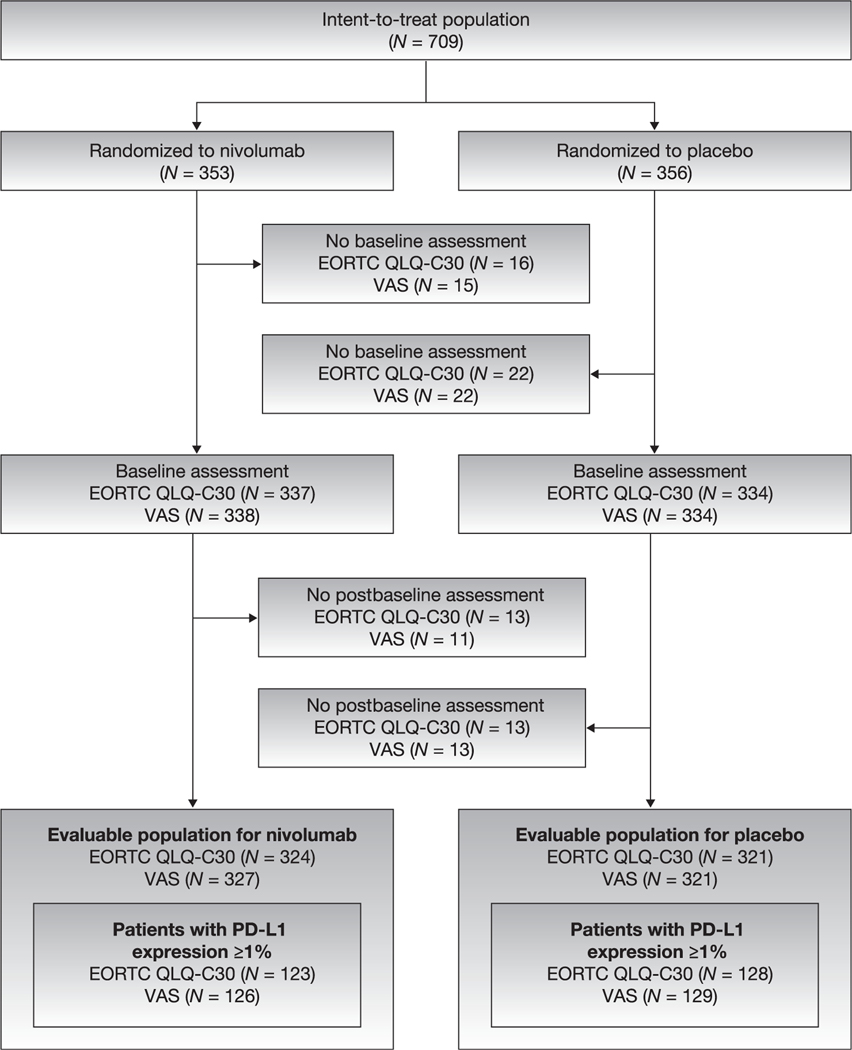
Patient disposition for the EORTC QLQ-C30 evaluable and VAS evaluable populations. EORTC QLQ-C30 = European Organisation for Research and Treatment of Cancer Quality of Life Questionnaire; PD-L1 = programmed death ligand 1; VAS = visual analog scale.

The overall EORTC QLQ-C30 evaluable population comprised 645 patients: 324 randomized to nivolumab and 321 randomized to placebo (Fig. 1). Tumor PD-L1 expression was ≥1% in 251 patients in the EORTC QLQ-C30 evaluable population. The two treatment arms were well balanced for demographic and baseline clinical characteristics (Table 1). Eastern Cooperative Oncology Group performance status was 2 for 2.2% of patients and 0 or 1 for other patients. Of the patients, 78.6% had cancer of the urinary bladder. Pathologic stage at resection was pT3 in 58.3% of patients, and 42.5% of patients had received cisplatin-based NAC for MIUC.

### 3.2. HRQoL at baseline and during treatment

For the ITT population, EORTC QLQ-C30 completion rates during treatment ranged from 85.0% to 95.5% for nivolumab and from 86.5% to 94.6% for placebo (Supplementary Table 1). The available data rate declined between baseline and week 49, from 95.5% to 38.5% in the nivolumab arm and from 93.8% to 36.2% in the placebo arm. The available data rate for the VAS also decreased during treatment. Similar trends in available data rate were observed for patients with PD-L1 expression ≥1% (Supplementary Table 2).

EORTC QLQ-C30 and VAS scores at baseline were generally comparable between treatment arms (Supplementary Table 3). Mean scores at baseline across all primary outcomes were comparable with those in general populations with similar age and gender distributions [34,35], except that the mean VAS score at baseline in the placebo arm (72.2) was worse than the score of 80.7 in the general population by more than the prespecified MID of 7 points. In patients with PD-L1 expression ≥1%, baseline mean VAS scores in both the nivolumab arm (72.3) and the placebo arm (70.8) were worse than the score in the general population by more than 7 points, and baseline mean EORTC QLQ-C30 role functioning in the nivolumab arm (77.2) was worse than the score of 84.1 in the general population by more than the prespecified MID of 6 points (Supplementary Table 4).

**Fig. 2 –:**
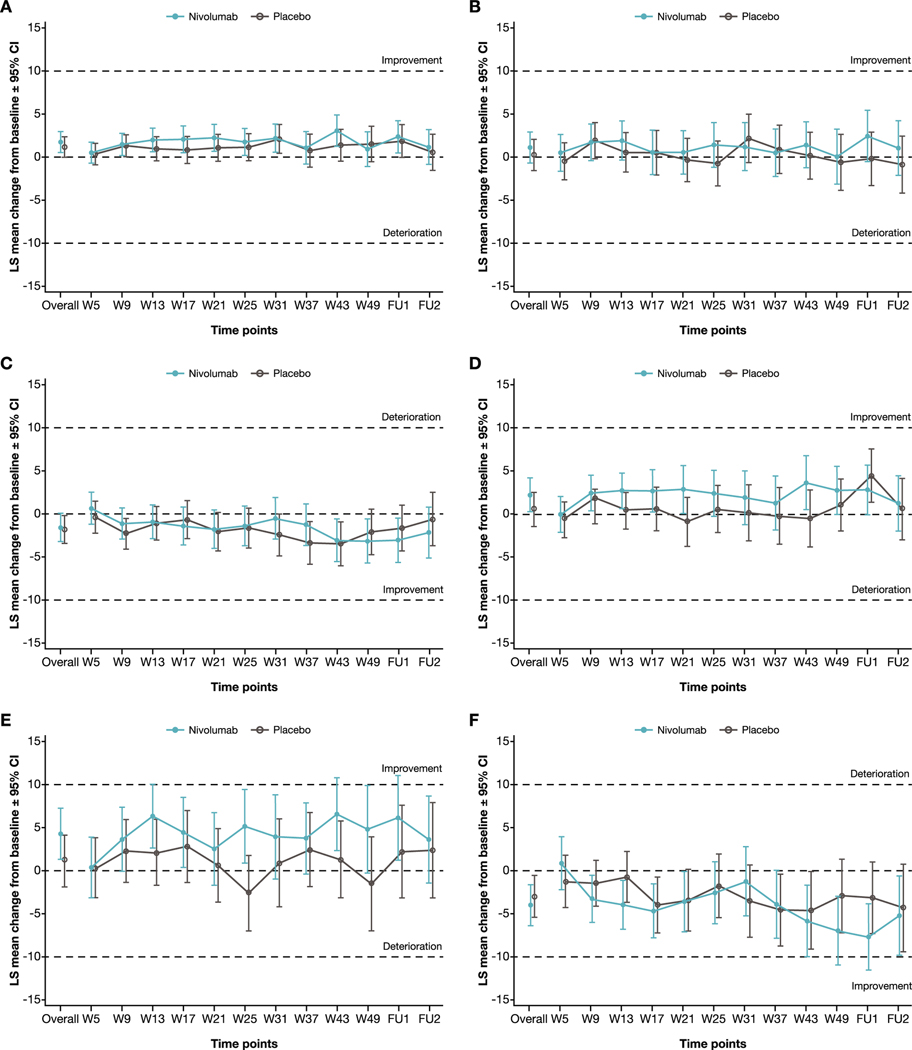
Linear mixed-effect model for repeated measures least squares mean change from baseline in HRQoL. (A) Physical functioning, (B) role functioning, and (C) fatigue for the EORTC QLQ-C30 evaluable population. (D) Physical functioning, (E) role functioning, and (F) fatigue for the EORTC QLQ-C30 evaluable population with PD-L1 expression ≥1%. CI = confidence interval; EORTC QLQ-C30 = European Organisation for Research and Treatment of Cancer Quality of Life Questionnaire; FU = follow-up; HRQoL = health-related quality of life; LS = least squares; W = week.

For both treatment arms, HRQoL was generally maintained during treatment in the overall EORTC QLQ-C30/VAS evaluable population and in patients with PD-L1 expression ≥1%. No clinically meaningful deterioration for EORTC QLQ-C30 global health status/QoL or VAS [19], or any of the other main outcomes (Fig. 2) was observed in patients treated with nivolumab or placebo. In the overall EORTC QLQ-C30/VAS evaluable population, nivolumab was noninferior to placebo on all the HRQoL outcomes based on LS mean change from baseline (Table 2). For patients with PD-L1 tumor expression ≥1%, noninferiority of nivolumab to placebo was not demonstrated for EORTC QLQ-C30 emotional functioning (the lower bound of the 95% confidence interval [CI] for the difference between nivolumab and placebo in LS mean change from baseline [−3.43] exceeded the prespecified noninferiority margin of −3; Supplementary Table 5). For the main and other outcomes, nivolumab was noninferior to placebo.

### 3.3. TTCD of HRQoL

**Fig. 3 –:**
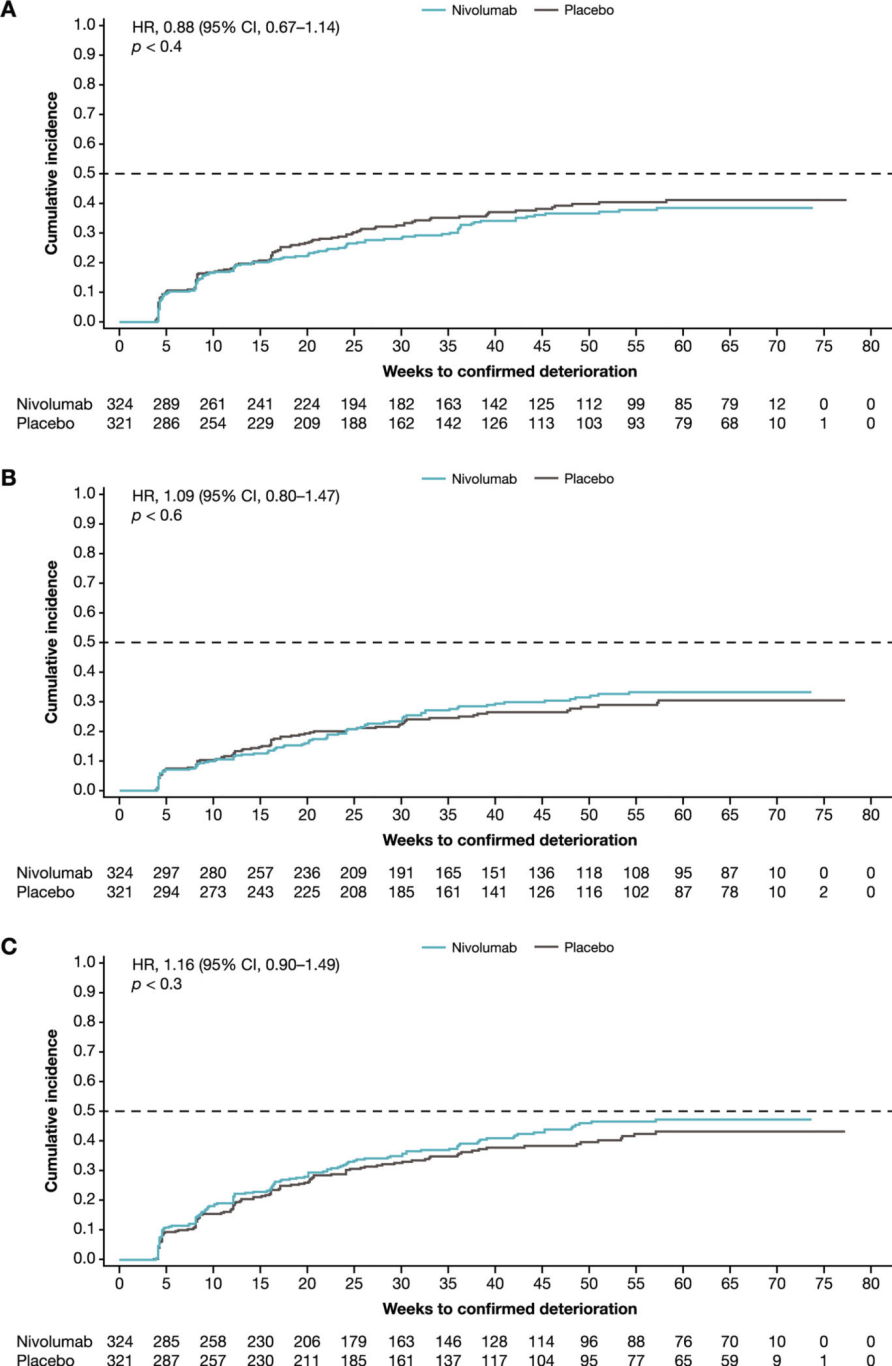

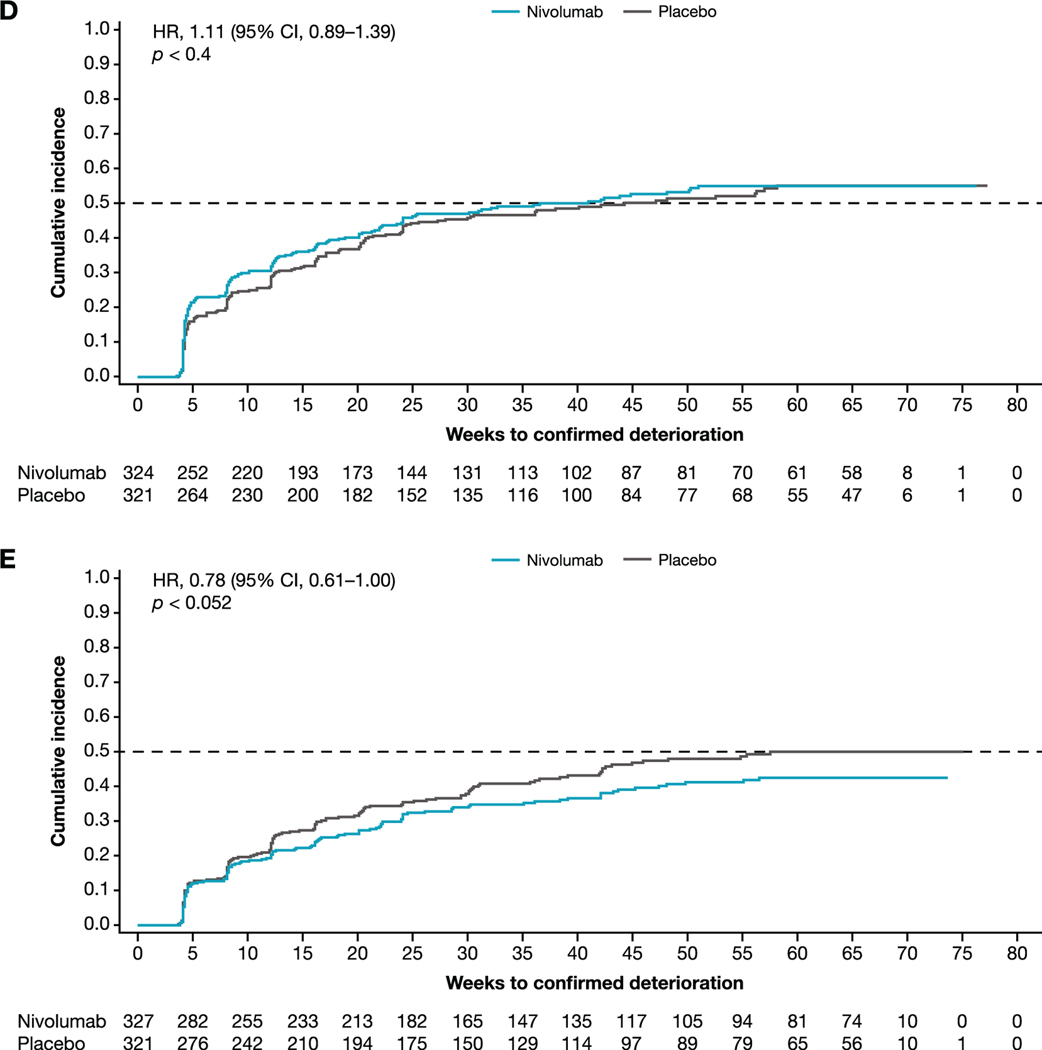
Time to confirmed deterioration of HRQoL: (A) global health status/QoL, (B) physical functioning, (C) role functioning, (D) fatigue, and (E) VAS. The analysis used the overall EORTC QLQ-C30 evaluable and VAS evaluable populations. CI = confidence interval; EORTC QLQ-C30 = European Organisation for Research and Treatment of Cancer Quality of Life Questionnaire; HR = hazard ratio; HRQoL = health-related quality of life; QoL = quality of life; VAS = visual analog scale.

Confirmed deterioration was defined as worsening above an a priori threshold of –10 points (EORTC QLQ-C30 global health status/QoL, physical functioning, and role functioning), +10 points (EORTC QLQ-C30 fatigue), or −7 points (VAS) at two or more consecutive visits. In both the full EORTC QLQ-C30 evaluable population (Fig. 3) and the patients with tumor PD-L1 expression ≥1% (Supplementary Fig. 1), the median TTCD of HRQoL was not reached for either nivolumab or placebo for EORTC QLQ-C30 global health status/QoL, physical functioning, or role functioning. For EORTC QLQ-C30 fatigue in the full EORTC QLQ-C30 evaluable population, the median TTCD was 41.0 wk for nivolumab and 44.3 wk for placebo (HR: 1.11, 95% CI, 0.89–1.39). For fatigue in patients with PD-L1 expression ≥1%, the median TTCD was 50.3 wk with nivolumab and 36.1 wk with placebo (HR: 0.97, 95% CI, 0.68–1.39).

For the VAS, the median TTCD was not reached with nivolumab. For placebo, it was 57.6 wk for the VAS evaluable population (HR: 0.78, 95% CI, 0.61–1.00) and 39.1 wk for patients with PD-L1 expression ≥1% (HR: 0.63, 95% CI, 0.42–0.93).

### 3.4. Risk of deterioration of HRQoL according to recurrence status

For all the main outcomes, risk of deterioration of HRQoL was significantly higher for patients with distant recurrence than for those with no recurrence (Table 3). For patients with local recurrence only, the risk of deterioration of EORTC QLQ-C30 global health status/QoL was significantly higher than that for patients with no recurrence. For the other main outcomes, the HRs were not significant.

## 4. Discussion

Historically, patients with MIUC who undergo radical cystectomy have often experienced decreased HRQoL. This can include impaired sexual function as a result of radical resection [36] or be a consequence of toxicities from NAC [21]. Moreover, Catto et al [37] recently reported that HRQoL after bladder cancer was worse than for other pelvic cancers. It is therefore vital not to further worsen HRQoL in these patients. In the present analysis based on the phase 3 CheckMate 274 trial, no clinically meaningful deterioration of HRQoL was observed during adjuvant treatment in either the full EORTC QLQ-C30/VAS evaluable population or patients with PD-L1 expression ≥1%. Nivolumab was noninferior to placebo on change in HRQoL during treatment, despite the higher rate of grade ≥3 treatment-related adverse events for nivolumab versus placebo (17.9% vs 7.2%) in CheckMate 274. By contrast, recurrence, especially distant recurrence, which was more prevalent in the placebo arm [19], was associated with worsening of HRQoL.

Our finding that adjuvant nivolumab did not worsen HRQoL is not without precedent. In another phase 3 randomized, double-blind, placebo-controlled trial (CheckMate 577), adjuvant nivolumab maintained HRQoL while increasing DFS in patients with resected esophageal or gastroesophageal junction cancer [38]. Moreover, in another phase 3 trial (CheckMate 238), adjuvant nivolumab prolonged recurrence-free survival in patients with resected stage III or IV melanoma, as compared with ipilimumab, without affecting HRQoL [39]. The value of assessing HRQoL in urothelial cancer is underscored by previous research linking better HRQoL with better prognosis in patients with locally advanced or metastatic bladder cancer [40]. However, it should be noted that baseline HRQoL in our study sample was comparable with that in age- and gender-matched general populations, which may have limited our ability to detect deteriorations in HRQoL scores during treatment. Moreover, a final evaluation of HRQoL in relation to efficacy in CheckMate 274 should consider data for overall survival, which are not yet available.

Limitations of the present study include assessment of HRQoL in <50% of patients from week 31 onward, largely because of patients discontinuing the study due to disease recurrence [19]. However, available data rates remained high (≥85%) throughout treatment. Another possible limitation is that the main MMRMs excluded observations after treatment discontinuation. As treatment discontinuation was mainly due to disease recurrence, drug toxicities, and adverse events unrelated to treatment [19], it is likely that the HRQoL estimates from this analysis were better than what would have been obtained if observations after treatment discontinuation had been included. However, the treatment arms had comparable proportions of treatment discontinuations [19], so the HRQoL analysis comparing nivolumab and placebo was unlikely to have been affected by missing observations. Moreover, in comparing nivolumab and placebo, the MIDs used were based on the conservative MID thresholds recently estimated by Cocks and colleagues [32] from a meta-analysis of published studies. In addition, the evaluation of noninferiority used the 95% CIs for between-group differences in overall LS mean changes from baseline. This approach makes it harder to demonstrate noninferiority with a smaller sample, because the 95% CIs will be wider and it is more likely that the upper or lower bound will exceed the MID. This may explain why noninferiority of nivolumab to placebo on emotional functioning was not demonstrated for patients with PD-L1 tumor expression ≥1%. Finally, only two follow-up EORTC QLQ-C30 assessments after recurrence were planned, and the rates of missing data for these follow-up assessments were high. Therefore, postrecurrence follow-up might not have been adequate to capture HRQoL deterioration at two or more consecutive assessments (as per the definition of confirmed deterioration) in some patients with recurrence.

## 5. Conclusions

Viewed together with the efficacy data from CheckMate 274, the present analysis indicates that the delay in recurrence after radical resection with nivolumab treatment may also delay or prevent deterioration of HRQoL. Nivolumab prolonged DFS in patients with MIUC without compromising HRQoL. The reproducibility of these findings should be confirmed with continued clinical research of nivolumab as an adjuvant treatment for MIUC.

## Supplementary Material

1

## Figures and Tables

**Table 1 –:** Demographic and baseline clinical characteristics

	Full EORTC QLQ-C30 evaluable population			Patients with PD-L1 expression ≥1%		
		
	Nivolumab (*N* = 324)	Placebo (*N* = 321)	Total (*N* = 645)	Nivolumab (*N* = 123)	Placebo (*N* = 128)	Total (*N* = 251)
Age (yr)						
Mean (SD)	65.4 (9.97)	65.8 (8.76)	65.6 (9.38)	64.6 (10.25)	66.1 (8.32)	65.4 (9.33)
Range	30–92	42–86	30–92	34–92	45–84	34–92
Sex, *n* (%)						
Female	79 (24.4)	75 (23.4)	154 (23.9)	33 (26.8)	27 (21.1)	60 (23.9)
Race, *n* (%)						
American Indian or Alaska Native	1 (0.3)	0	1 (0.2)	1 (0.8)	0	1 (0.4)
Asian	73 (22.5)	72 (22.4)	145 (22.5)	28 (22.8)	27 (21.1)	55 (21.9)
Black or African American	2 (0.6)	2 (0.6)	4 (0.6)	0	2 (1.6)	2 (0.8)
White	244 (75.3)	241 (75.1)	485 (75.2)	94 (76.4)	96 (75.0)	190(75.7)
Other	4 (1.2)	5 (1.6)	9 (1.4)	0	2 (1.6)	2 (0.8)
Missing	0	1 (0.3)	1 (0.2)	0	1 (0.8)	1 (0.4)
Weight (kg)						
Mean (SD)	73.4 (15.65)	73.4 (14.55)	73.4 (15.10)	73.5 (16.12)	74.8 (14.69)	74.2 (15.39)
Smoking status, *n* (%)						
Former/current smoker	220 (67.9)	222 (69.2)	442 (68.5)	86 (69.9)	91 (71.1)	177 (70.5)
Never smoker	102 (31.5)	96 (29.9)	198 (30.7)	36 (29.3)	36 (28.1)	72 (28.7)
Unknown/not reported	2 (0.6)	3 (0.9)	5 (0.8)	1 (0.8)	1 (0.8)	2 (0.8)
ECOG performance status, *n* (%)						
0	207 (63.9)	207 (64.5)	414 (64.2)	76 (61.8)	80 (62.5)	156 (62.2)
1	112 (34.6)	105 (32.7)	217 (33.6)	45 (36.6)	44 (34.4)	89 (35.5)
2	5 (1.5)	9 (2.8)	14 (2.2)	2 (1.6)	4(3.1)	6 (2.4)
Tumor type, *n* (%)						
Urinary bladder	258 (79.6)	249 (77.6)	507 (78.6)	102 (82.9)	104 (81.3)	206(82.1)
Renal pelvis	38 (11.7)	49 (15.3)	87 (13.5)	15 (12.2)	13 (10.2)	28 (11.2)
Ureter	28 (8.6)	23 (7.2)	51 (7.9)	6 (4.9)	11 (8.6)	17 (6.8)
Time from diagnosis to randomization (yr), *n* (%)						
<1	296 (91.4)	295 (91.9)	591 (91.6)	115 (93.5)	116 (90.6)	231 (92.0)
≥1	28 (8.6)	26 (8.1)	54 (8.4)	8 (6.5)	12 (9.4)	20 (8.0)
Pathologic stage at resection, *n* (%)						
<pT2	15 (4.6)	16 (5.0)	31 (4.8)	5 (4.1)	4(3.1)	9 (3.6)
pT2	56 (17.3)	61 (19.0)	117 (18.1)	16 (13.0)	25 (19.5)	41 (16.3)
pT3	194 (59.9)	182 (56.7)	376 (58.3)	78 (63.4)	73 (57.0)	151 (60.2)
pT4a	52 (16.0)	58 (18.1)	110 (17.1)	21 (l7.l)	25 (19.5)	46 (18.3)
pTx	4(1.2)	0	4 (0.6)	3 (2.4)	0	3 (1.2)
pTis	2 (0.6)	3 (0.9)	5 (0.8)	0	0	0
Missing	1 (0.3)	1 (0.3)	2 (0.3)	0	1 (0.8)	1 (0.4)
PD-L1 expression level, *n* (%)						
<1%	199 (61.4)	188 (58.6)	387 (60.0)	0	1 (0.8)	1 (0.4)
≥1%	122 (37.7)	127 (39.6)	249 (38.6)	122 (99.2)	126 (98.4)	248 (98.8)
Missing	3 (0.9)	6(1.9)	9 (1.4)	1 (0.8)	1 (0.8)	2 (0.8)
Receipt of neoadjuvant cisplatin-based chemotherapy for MIUC, *n* (%)						
Yes	137 (42.3)	137 (42.7)	274 (42.5)	51 (41.5)	52 (40.6)	103 (41.0)
No	187 (57.7)	184 (57.3)	371 (57.5)	72 (58.5)	76 (59.4)	148 (59.0)
Pathologic nodal status, *n* (%)						
N+	135 (41.7)	133 (41.4)	268 (41.6)	47 (38.2)	49 (38.3)	96 (38.2)
Nx or N0 with <10 nodes removed	l04(32.l)	102 (31.8)	206 (31.9)	38 (30.9)	41 (32.0)	79 (31.5)
N0 with ≥10 nodes removed	85 (26.2)	86 (26.8)	171 (26.5)	38 (30.9)	38 (29.7)	76 (30.3)

ECOG = Eastern Cooperative Oncology Group; EORTC QLQ-C30 = European Organisation for Research and Treatment of Cancer Quality of Life Questionnaire; MIUC = muscle-invasive urothelial carcinoma; PD-L1 = programmed death ligand 1; SD = standard deviation.

**Table 2 –:** Linear mixed-effect model for repeated measure analysis of change from baseline for nivolumab versus placebo^a^

	LS mean change from baseline (95% CI)			Prespecified noninferiority margin, MID
		
	Nivolumab	Placebo	Difference ^b^	
*Main outcomes*				
EORTC QLQ-C30				
Global health status/QoL	1.80 (0.28–3.31)	1.69 (0.17–3.22)	0.10 (−2.00 to 2.20)	−4
Physical functioning	1.68 (0.46–2.89)	1.10 (−0.12 to 2.32)	0.58 (−1.11 to 2.26)	−5
Role functioning	1.05 (−0.75 to 2.86)	0.19 (−1.63 to 2.01)	0.87 (−1.64 to 3.37)	−6
Fatigue	−1.58 (−3.20 to 0.03)	−1.80 (−3.43 to −0.17)	0.22 (−2.02 to 2.46)	+5
EQ-5D-3L				
VAS	1.43 (−0.28 to 3.13)	−0.73 (−2.47 to 1.01)	2.15 (−0.23 to 4.54)	−7
*Other outcomes*				
Emotional functioning	1.73 (0.34–3.12)	2.23 (0.83–3.63)	−0.50 (−2.43 to 1.43)	−3
Cognitive functioning	−0.95 (−2.27 to 0.37)	−2.23 (−3.56 to −0.91)	1.28 (−0.55 to 3.11)	−3
Social functioning	3.21 (1.62–4.81)	3.68 (2.07–5.29)	−0.46 (−2.68 to 1.76)	−5
Nausea/vomiting	0.84 (0.17–1.51)	−0.22 (−0.90 to 0.46)	1.06 (0.13–2.00)	+3
Pain	0.87 (−0.86 to 2.61)	1.44 (−0.30 to 3.19)	−0.57 (−2.97 to 1.83)	+6
Dyspnea	1.08 (−0.57 to 2.74)	0.76 (−0.91 to 2.43)	0.32 (−1.98 to 2.62)	+4
Insomnia	−2.42 (−4.48 to −0.37)	−3.08 (−5.15 to −1.01)	0.67 (−2.19 to 3.50)	+4
Appetite loss	−0.38 (−1.87 to 1.11)	−2.84 (−4.35 to −1.33)	2.46 (0.39−4.53)	+5
Constipation	−4.10 (−5.78 to −2.42)	−2.05 (−3.74 to −0.35)	−2.05 (−4.38 to 0.28)	+5
Diarrhea	1.45 (0.20–2.70)	0.37 (−0.90 to 1.63)	1.08 (−0.66 to 2.82)	+3
Financial difficulties	−3.61 (−5.36 to −1.87)	−2.49 (−4.24 to −0.73)	−1.12 (−3.54 to 1.30)	+3

CI = confidence interval; EORTC QLQ-C30 = European Organisation for Research and Treatment of Cancer Quality of Life Questionnaire; LS = least squares; MID = minimally important difference; QoL = quality of life; VAS = visual analog scale.

a The analysis used the overall EORTC QLQ-C30 evaluable and VAS evaluable populations.

b Noninferiority: upper bound (EORTC QLQ-C30 symptom domains) or lower bound (other outcomes) of 95% CI of the overall LS mean difference does not exceed the prespecified noninferiority margin.

**Table 3 –:** Risk of deterioration of HRQoL according to recurrence status^a^

	Any recurrence	Local recurrence only	Distant recurrence
EORTC QLQ-C30 (*N* = 645)			
Recurrence, *n* (%)	210 (32.6)	74 (11.5)	136 (21.1)
Adjusted HR (95% CI) for recurrence vs no recurrence			
Global health status/QoL	3.4 (2.3–5.3)	3.0 (1.6–5.6)	3.8 (2.3–6.3)
Physical functioning	3.9 (2.6–6.0)	1.6 (0.7–3.7)	5.8 (3.7–9.1)
Role functioning	2.8 (1.9–4.2)	1.7 (0.9–3.5)	3.6 (2.3–5.7)
Fatigue	1.6 (1.1–2.5)	1.2 (0.6–2.0)	2.0 (1.2–3.3)
EQ-5D-3L (*N* = 648)			
Recurrence, *n* (%)	212 (32.7)	75 (11.6)	137 (21.1)
Adjusted HR (95% CI) for recurrence vs no recurrence			
VAS	1.9 (1.2–3.0)	1.3 (0.6–2.8)	2.4 (1.4–4.1)

CI = confidence interval; EORTC QLQ-C30 = European Organisation for Research and Treatment of Cancer Quality of Life Questionnaire; HR = hazard ratio; HRQoL = health-related quality of life; VAS = visual analog scale.

a Hazard ratios were calculated with no recurrence as the reference category.
