# Supplementary material for: Health-related Quality of Life with Adjuvant Nivolumab After Radical Resection for High-risk Muscle-invasive Urothelial Carcinoma: Results from the Phase 3 CheckMate 274 Trial
Source: Eur Urol Oncol. Author manuscript; Available in PMC 2023 Mar 30. (PMC10062393; doi:10.1016/j.euo.2022.02.003)

# Supplementary Material

**Supplementary Table 1 – Available data and completion rates for the EORTC** **QLQ-C30 and VAS**

| **Time point** | **EORTC QLQ-C30** | | | | **VAS** | | | |
| --- | --- | --- | --- | --- | --- | --- | --- | --- |
|  | **Completion rate** | | **Available data rate** | | **Completion rate** | | **Available data rate** | |
|  | **Nivolumab (*N* = 353)**  ***n/n* (%)** | **Placebo (*N* = 356)**  ***n/n* (%)** | **Nivolumab (*N* = 353)**  ***n/n* (%)** | **Placebo (*N* = 356)**  ***n/n* (%)** | **Nivolumab (*N* = 353)**  ***n/n* (%)** | **Placebo (*N* = 356)**  ***n/n* (%)** | **Nivolumab (*N* = 353)**  ***n/n* (%)** | **Placebo (*N* = 356)**  ***n/n* (%)** |
| Baseline | 337/353 (95.5) | 334/356 (93.8) | 337/353 (95.5) | 334/356 (93.8) | 338/353 (95.8) | 334/356 (93.8) | 338/353 (95.8) | 334/356 (93.8) |
| Week 5 | 308/328 (93.9) | 307/329 (93.3) | 308/353 (87.3) | 307/356 (86.2) | 305/328 (93.0) | 303/329 (92.1) | 305/353 (86.4) | 303/356 (85.1) |
| Week 9 | 286/308 (92.9) | 297/314 (94.6) | 286/353 (81.0) | 297/356 (83.4) | 287/308 (93.2) | 294/314 (93.6) | 287/353 (81.3) | 294/356 (82.6) |
| Week 13 | 260/286 (90.9) | 254/277 (91.7) | 260/353 (73.7) | 254/356 (71.3) | 262/286 (91.6) | 255/277 (92.1) | 262/353 (74.2) | 255/356 (71.6) |
| Week 17 | 228/255 (89.4) | 221/253 (87.4) | 228/353 (64.6) | 221/356 (62.1) | 229/255 (89.8) | 220/253 (87.0) | 229/353 (64.9) | 220/356 (61.8) |
| Week 21 | 217/244 (88.9) | 223/241 (92.5) | 217/353 (61.5) | 223/356 (62.6) | 218/244 (89.3) | 226/241 (93.8) | 218/353 (61.8) | 226/356 (63.5) |
| Week 25 | 198/222 (89.2) | 198/218 (90.8) | 198/353 (56.1) | 198/356 (55.6) | 200/222 (90.1) | 198/218 (90.8) | 200/353 (56.7) | 198/356 (55.6) |
| Week 31 | 175/206 (85.0) | 169/194 (87.1) | 175/353 (49.6) | 169/356 (47.5) | 175/206 (85.0) | 169/194 (87.1) | 175/353 (49.6) | 169/356 (47.5) |
| Week 37 | 161/183 (88.0) | 156/173 (90.2) | 161/353 (45.6) | 156/356 (43.8) | 159/183 (86.9) | 156/173 (90.2) | 159/353 (45.0) | 156/356 (43.8) |
| Week 43 | 146/169 (86.4) | 135/156 (86.5) | 146/353 (41.4) | 135/356 (37.9) | 146/169 (86.4) | 135/156 (86.5) | 146/353 (41.4) | 135/356 (37.9) |
| Week 49 | 136/151 (90.1) | 129/139 (92.8) | 136/353 (38.5) | 129/356 (36.2) | 134/151 (88.7) | 130/139 (93.5) | 134/353 (38.0) | 130/356 (36.5) |
| Follow-up visit 1 | 251/316 (79.4) | 234/299 (78.3) | 251/353 (71.1) | 234/356 (65.7) | 250/316 (79.1) | 235/299 (78.6) | 250/353 (70.8) | 235/356 (66.0) |
| Follow-up visit 2 | 212/284 (74.6) | 199/257 (77.4) | 212/353 (60.1) | 199/356 (55.9) | 221/284 (77.8) | 197/257 (76.7) | 221/353 (62.6) | 197/356 (55.3) |

The analysis used the intention-to-treat population.

EORTC QLQ-C30 = European Organisation for Research and Treatment of Cancer Quality of Life Questionnaire; *n/n* = no. of responders/no. at risk; VAS = visual analog scale.

**Supplementary Table 2 – Available data rates for the EORTC** **QLQ-C30 and VAS for patients with PD-L1 expression ≥1%**

|  | **EORTC QLQ-C30** | | **VAS** | |
| --- | --- | --- | --- | --- |
| **Time point** | **Nivolumab (*N* = 140)**  ***n/n* (%)** | **Placebo\| (*N* = 142)**  ***n/n* (%)** | **Nivolumab (*N* = 140)**  ***n/n* (%)** | **Placebo (*N* = 142)**  ***n/n* (%)** |
| Baseline | 130/140 (92.9%) | 136/142 (95.8%) | 132/140 (94.3%) | 137/142 (96.5%) |
| Week 5 | 122/140 (87.1%) | 121/142 (85.2%) | 122/140 (87.1%) | 120/142 (84.5%) |
| Week 9 | 113/140 (80.7%) | 114/142 (80.3%) | 114/140 (81.4%) | 112/142 (78.9%) |
| Week 13 | 104/140 (74.3%) | 91/142 (64.1%) | 105/140 (75.0%) | 91/142 (64.1%) |
| Week 17 | 91/140 (65.0%) | 79/142 (55.6%) | 92/140 (65.7%) | 78/142 (54.9%) |
| Week 21 | 83/140 (59.3%) | 79/142 (55.6%) | 83/140 (59.3%) | 82/142 (57.7%) |
| Week 25 | 77/140 (55.0%) | 70/142 (49.3%) | 79/140 (56.4%) | 69/142 (48.6%) |
| Week 31 | 70/140 (50.0%) | 60/142 (42.3%) | 70/140 (50.0%) | 59/142 (41.5%) |
| Week 37 | 65/140 (46.4%) | 56/142 (39.4%) | 64/140 (45.7%) | 56/142 (39.4%) |
| Week 43 | 60/140 (42.9%) | 48/142 (33.8%) | 60/140 (42.9%) | 48/142 (33.8%) |
| Week 49 | 52/140 (37.1%) | 42/142 (29.6%) | 52/140 (37.1%) | 42/142 (29.6%) |
| Follow-up visit 1 | 98/140 (70.0%) | 93/142 (65.5%) | 98/140 (70.0%) | 92/142 (64.8%) |
| Follow-up visit 2 | 83/140 (59.3%) | 81/142 (57.0%) | 89/140 (63.6%) | 79/142 (55.6%) |

EORTC QLQ-C30 = European Organisation for Research and Treatment of Cancer Quality of Life Questionnaire; *n/n* = no. of responders/no. at risk; PD-L1 = programmed death ligand 1; VAS = visual analog scale.

**Supplementary Table 3 – Mean HRQoL domain scores at baseline (EORTC QLQ-C30 evaluable population)**

|  | **Nivolumab**  **(*N* = 324)** | **Placebo**  **(*N* = 321)** | **Overall**  **(*N* = 645)** | **General population norm ^a^** |
| --- | --- | --- | --- | --- |
| **Main outcomes** |  |  |  |  |
| EORTC QLQ-C30, mean, (SD) |  |  |  |  |
| Global health status/QoL | 71.1 (20.2) | 70.7 (20.9) | 70.9 (20.5) | 65.8 |
| Physical functioning | 83.8 (14.8) | 83.3 (16.3) | 83.5 (15.6) | 84.4 |
| Role functioning | 81.1 (24.2) | 81.8 (25.0) | 81.5 (24.6) | 84.1 |
| Fatigue | 24.7 (20.2) | 24.3 (21.1) | 24.5 (20.6) | 26.9 |
| EQ-5D-3L |  |  |  |  |
| VAS | 75.6 (19.3) | 72.2 (23.7) | 73.9 (21.7) | 80.7 |
| **Other outcomes** |  |  |  |  |
| EORTC QLQ-C30, mean (SD) |  |  |  |  |
| Emotional functioning | 85.2 (16.6) | 82.0 (20.6) | 83.6 (18.8) | 77.4 |
| Cognitive functioning | 89.7 (15.3) | 89.6 (16.0) | 89.6 (15.6) | 86.4 |
| Social functioning | 84.4 (21.0) | 80.6 (24.4) | 82.5 (22.8) | 87.5 |
| Nausea/vomiting | 1.8 (6.9) | 2.8 (8.7) | 2.3 (7.9) | 3.6 |
| Pain | 15.1 (20.2) | 14.8 (20.8) | 15.0 (20.5) | 23.9 |
| Dyspnea | 11.3 (20.9) | 9.4 (17.6) | 10.4 (19.3) | 16.2 |
| Insomnia | 21.9 (26.9) | 21.5 (27.7) | 21.7 (27.3) | 26.7 |
| Appetite loss | 10.1 (20.4) | 10.8 (22.1) | 10.5 (21.2) | 7.7 |
| Constipation | 16.3 (24.2) | 16.9 (25.0) | 16.6 (24.6) | 10.8 |
| Diarrhea | 5.2 (13.7) | 6.1 (16.3) | 5.7 (15.0) | 7.6 |
| Financial difficulties | 16.1 (24.5) | 16.8 (26.6) | 16.5 (25.5) | 9.4 |

EORTC QLQ-C30 = European Organisation for Research and Treatment of Cancer Quality of Life Questionnaire; HRQoL = health-related quality of life; VAS = visual analog scale.

^a^ The EORTC QLQ-C30 norm scores are from the European general population data based on 11 European Union countries (*N* = 11,343) [1], reweighted by the EORTC QLQ-C30 evaluable population’s age and sex distributions. EQ-5D norm scores are from the UK general population (*N* = 3,395) [2]. Higher scores for EORTC QLQ-C30 symptom domains indicate a worse level of symptomatology.

**References**

[1] Nolte S, Liegl G, Petersen MA, et al. General population normative data for the EORTC QLQ-C30 health-related quality of life questionnaire based on 15,386 persons across 13 European countries, Canada and the Unites States. Eur J Cancer 2019;107:153-63.

[2] Szende A, Janssen B. Population norms for the EQ-5D. In: Szende A, Janssen B, Cabases J, editors. Self-Reported Population Health: An International Perspective based on EQ-5D Dordrecht: Springer; 2014. p. 19-30.

**Supplementary Table 4 – Mean HRQoL domain scores at baseline (EORTC QLQ-C30 evaluable population with PD-L1 expression ≥1%)**

|  | **Nivolumab**  **(*N* = 123)** | **Placebo**  **(*N* = 128)** | **Overall**  **(*N* = 251)** | **General population norm ^a^** |
| --- | --- | --- | --- | --- |
| **Main outcomes** |  |  |  |  |
| EORTC QLQ-C30, mean (SD) |  |  |  |  |
| Global health status/QoL | 67.3 (22.3) | 68.6 (20.4) | 68.0 (21.3) | 65.8 |
| Physical functioning | 83.7 (14.4) | 82.6 (15.5) | 83.1 (14.9) | 84.4 |
| Role functioning | 77.2 (25.8) | 78.5 (26.6) | 77.9 (26.2) | 84.1 |
| Fatigue | 25.7 (19.9) | 26.2 (22.5) | 25.9 (21.3) | 26.9 |
| EQ-5D-3L |  |  |  |  |
| VAS | 72.3 (21.0) | 70.8 (23.5) | 71.5 (22.3) | 80.7 |
| **Other outcomes** |  |  |  |  |
| EORTC QLQ-C30, mean (SD) |  |  |  |  |
| Emotional functioning | 83.9 (18.7) | 82.3 (17.1) | 83.0 (17.9) | 77.4 |
| Cognitive functioning | 89.3 (15.6) | 88.2 (16.6) | 88.7 (16.0) | 86.4 |
| Social functioning | 81.8 (22.9) | 78.2 (25.5) | 80.0 (24.3) | 87.5 |
| Nausea/vomiting | 2.0 (6.9) | 3.5 (10.8) | 2.8 (9.1) | 3.6 |
| Pain | 15.4 (19.8) | 15.9 (21.9) | 15.7 (20.9) | 23.9 |
| Dyspnea | 11.4 (19.9) | 11.3 (19.4) | 11.3 (19.6) | 16.2 |
| Insomnia | 26.0 (29.1) | 21.4 (27.4) | 23.6 (28.3) | 26.7 |
| Appetite loss | 13.1 (22.5) | 10.4 (21.6) | 11.7 (22.1) | 7.7 |
| Constipation | 18.0 (25.8) | 17.8 (24.8) | 17.9 (25.2) | 10.8 |
| Diarrhea | 7.4 (15.8) | 6.0 (16.0) | 6.7 (15.8) | 7.6 |
| Financial difficulties | 16.8 (23.5) | 20.2 (28.8) | 18.5 (26.4) | 9.4 |

EORTC QLQ-C30 = European Organisation for Research and Treatment of Cancer Quality of Life Questionnaire; HRQoL = health-related quality of life; PD-L1 = programmed death ligand 1; VAS = visual analog scale.

^a^ The EORTC QLQ-C30 norm scores are from the European general population data based on 11 European Union countries (*N* = 11,343) [1], reweighted by the EORTC QLQ-C30 evaluable population’s age and sex distributions. EQ-5D norm scores are from the UK general population (*N* = 3,395) [2]. Higher scores for EORTC QLQ-C30 symptom domains indicate a worse level of symptomatology.

**References**

[1] Nolte S, Liegl G, Petersen MA, et al. General population normative data for the EORTC QLQ-C30 health-related quality of life questionnaire based on 15,386 persons across 13 European countries, Canada and the Unites States. Eur J Cancer 2019;107:153-63.

[2] Szende A, Janssen B. Population norms for the EQ-5D. In: Szende A, Janssen B, Cabases J, editors. Self-Reported Population Health: An International Perspective based on EQ-5D Dordrecht: Springer; 2014. p. 19-30.

**Supplementary Table 5 – Linear mixed-effect model for repeated measures analysis of change from baseline for nivolumab versus placebo in patients with PD-L1 expression ≥1%**

|  | **LS mean change from baseline (95% CI)** | | | **Prespecified noninferiority margin** |
| --- | --- | --- | --- | --- |
|  | **Nivolumab** | **Placebo** | **Difference ^a^** |  |
| **Main outcomes** |  |  |  |  |
| EORTC QLQ-C30 |  |  |  |  |
| Global health status/QoL | 4.44 (2.07–6.82) | 2.43 (0.01–4.85) | 2.01 (–1.34 to 5.36) | –4 |
| Physical functioning | 2.22 (0.26–4.18) | 0.53 (–1.47 to 2.52) | 1.69 (–1.07 to 4.46) | –5 |
| Role functioning | 4.24 (1.28–7.20) | 1.08 (–1.92 to 4.08) | 3.16 (–1.00 to 7.32) | –6 |
| Fatigue | –4.03 (–6.40 to –1.66) | –3.00 (–5.42 to –0.58) | –1.03 (–4.38 to 2.32) | +5 |
| EQ-5D-3L |  |  |  |  |
| VAS | 2.88 (–0.09 to 5.85) | –1.35 (–4.43 to 1.73) | 4.24 (0.01–8.46) | –7 |
| **Other outcomes** |  |  |  |  |
| EORTC QLQ-C30 |  |  |  |  |
| Emotional functioning | 2.87 (0.82–4.93) | 3.41 (1.32–5.50) | –0.54 (–3.43 to 2.36) | –3 |
| Cognitive functioning | –0.51 (–2.54 to 1.51) | –1.60 (–3.66 to 0.45) | 1.09 (–1.77 to 3.95) | –3 |
| Social functioning | 4.94 (2.53–7.35) | 3.57 (1.12–6.01) | 1.37 (–2.03 to 4.77) | –5 |
| Nausea/vomiting | 0.47 (–0.63 to 1.56) | –0.09 (–1.20 to 1.03) | 0.55 (–0.99 to 2.10) | +3 |
| Pain | –0.03 (–2.95 to 2.90) | 3.29 (0.31–6.27) | –3.31 (–7.44 to 0.82) | +6 |
| Dyspnea | –1.66 (–4.23 to 0.92) | 0.94 (–1.69 to 3.58) | –2.60 (–6.24 to 1.04) | +4 |
| Insomnia | –7.60 (–10.90 to –4.30) | –4.92 (–8.29 to –1.55) | –2.68 (–7.36 to 1.99) | +4 |
| Appetite loss | –2.72 (–4.64 to –0.81) | –4.01 (–5.97 to –2.05) | 1.29 (–1.43 to 4.00) | +5 |
| Constipation | –6.55 (–9.36 to –3.74) | –3.12 (–5.97 to –0.26) | –3.44 (–7.39 to 0.52) | +5 |
| Diarrhea | 0.04 (–1.99 to 2.07) | –0.06 (–2.12 to 2.00) | 0.10 (–2.76 to 2.97) | +3 |
| Financial difficulties | –4.87 (–7.70 to –2.04) | –3.79 (–6.67 to –0.91) | –1.08 (–5.07 to 2.92) | +3 |

CI = confidence interval; EORTC QLQ-C30 = European Organisation for Research and Treatment of Cancer Quality of Life Questionnaire; LS = least squares; PD-L1 = programmed death ligand 1; VAS = visual analog scale.

^a^ Noninferiority: upper bound (fatigue) or lower bound (other outcomes) of 95% CI of the overall LS mean difference does not exceed the prespecified noninferiority margin.

**Supplementary Fig. 1 – Time to confirmed deterioration of HRQoL in patients with PD-L1 expression ≥1%. (A)** Global health status/QoL. **(B)** Physical functioning. **(C)** Role functioning. **(D)** Fatigue. **(E)** VAS. CI = confidence interval; HR = hazard ratio; HRQoL = health-related quality of life; PD-L1 = programmed death ligand 1; VAS = visual analog scale.


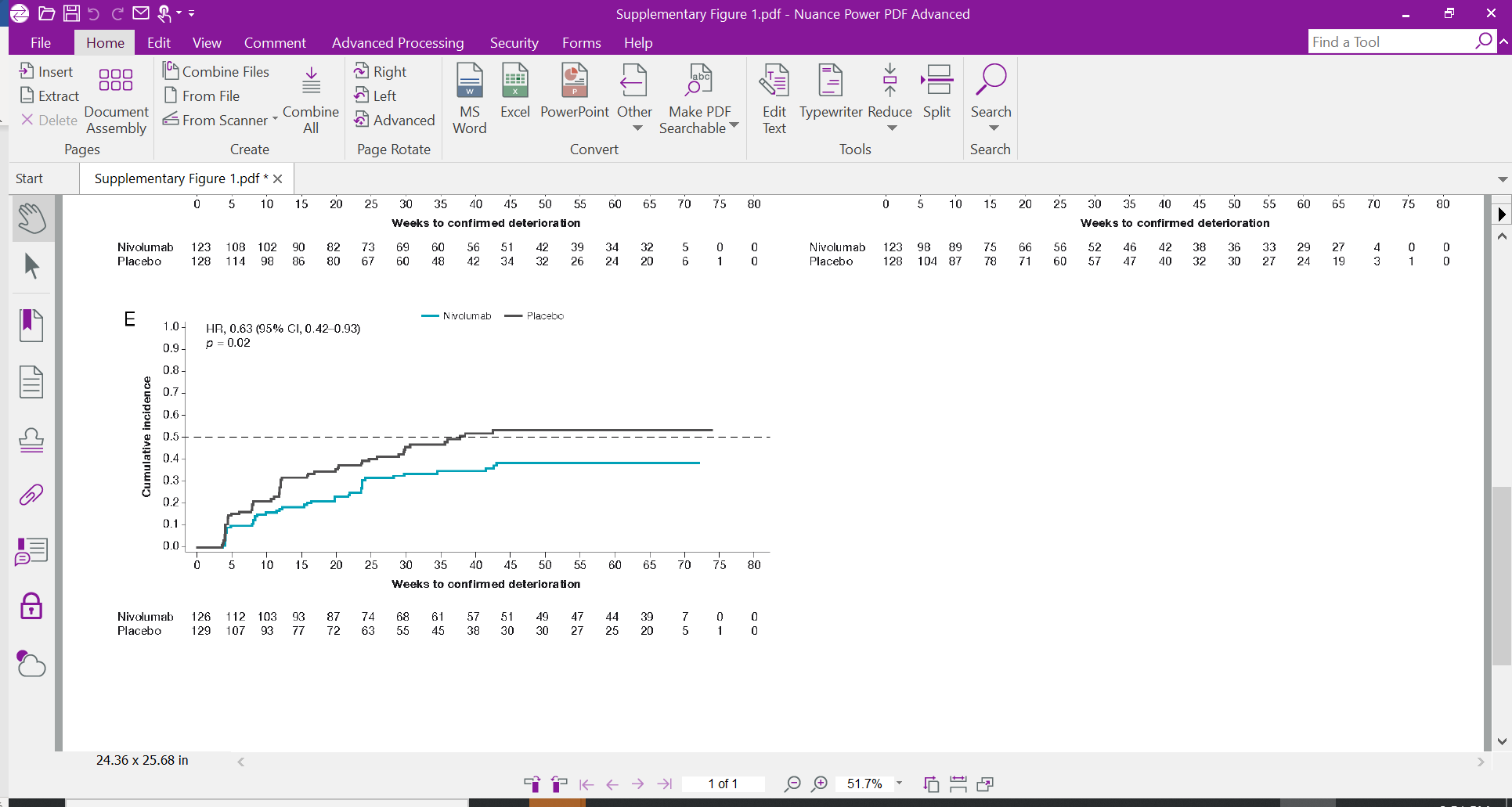

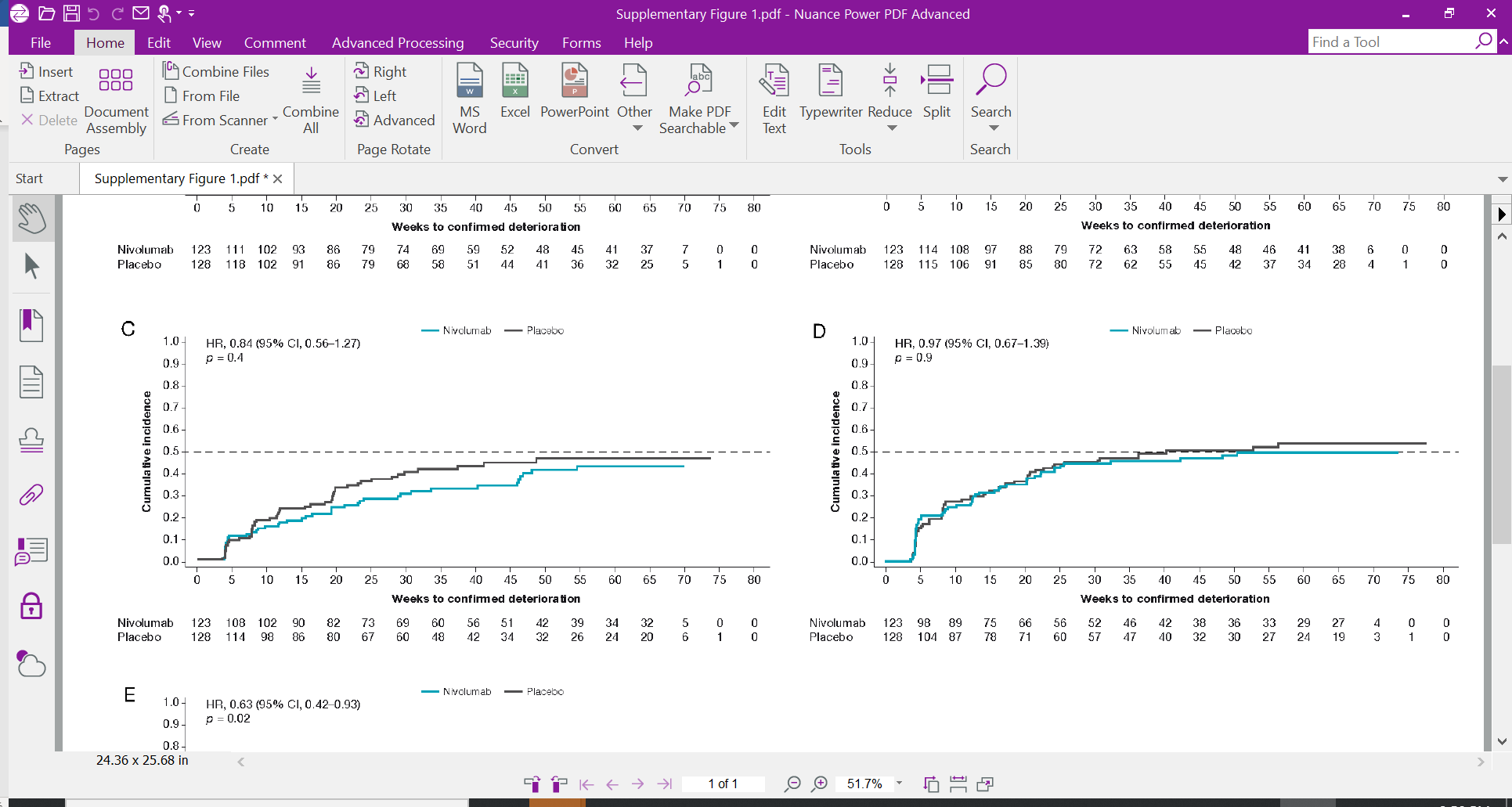

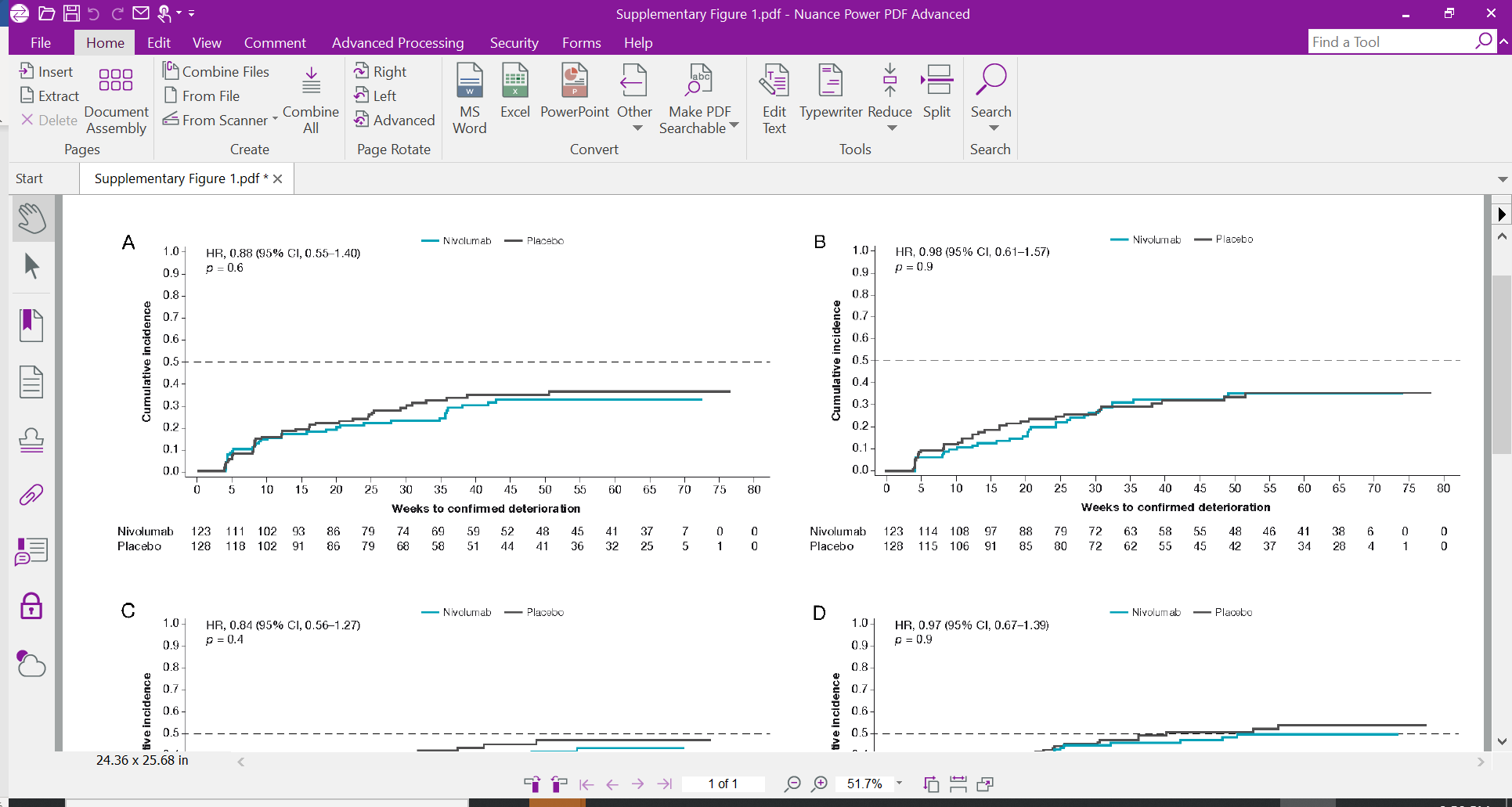

Supplement: 1 [file NIHMS1868090-supplement-1.docx]
